# The Functional Meaning of 5′UTR in Protein-Coding Genes

**DOI:** 10.3390/ijms24032976

**Published:** 2023-02-03

**Authors:** Natalia Ryczek, Aneta Łyś, Izabela Makałowska

**Affiliations:** Institute of Human Biology and Evolution, Adam Mickiewicz University in Poznań, Uniwersytetu Ponańskiego 6, 61-614 Poznań, Poland

**Keywords:** 5′UTR, protein-coding genes, uORF, head-to-head genes overlap, miRNA

## Abstract

As it is well known, messenger RNA has many regulatory regions along its sequence length. One of them is the 5′ untranslated region (5’UTR), which itself contains many regulatory elements such as upstream ORFs (uORFs), internal ribosome entry sites (IRESs), microRNA binding sites, and structural components involved in the regulation of mRNA stability, pre-mRNA splicing, and translation initiation. Activation of the alternative, more upstream transcription start site leads to an extension of 5′UTR. One of the consequences of 5′UTRs extension may be head-to-head gene overlap. This review describes elements in 5′UTR of protein-coding transcripts and the functional significance of protein-coding genes 5′ overlap with implications for transcription, translation, and disease.

## 1. Introduction

The 5′ untranslated region is the mRNA domain that contains plenty of elements such as upstream ORFs (uORFs), internal ribosome entry sites, microRNA binding sites, and structural components involved in the regulation of mRNA stability, pre-mRNA splicing, and translation initiation. Deregulation of cis-regulatory elements or secondary structures within the 5′UTRs may cause a change in gene expression [1]. This shows the functional importance of 5′UTR in control of gene expression. Furthermore, a growing number of evidence demonstrates that mutations within 5′UTRs are often linked with diseases, including cancer [2,3,4]. Oncogenes and tumor suppressors require precise regulation and often express transcripts containing various and atypically long 5′ untranslated regions with new regulatory elements, such as uORFs or secondary structures [5,6]. Moreover, extension of 5′ UTR due to the use of an alternative promoter may lead to an overlap with a gene located on the opposite DNA strand. Such overlap at 5’ ends may be associated with additional regulatory functions [7,8]. This review summarizes the roles of 5’UTRs and the regulatory mechanisms in which these sequences are involved.

## 2. Upstream Open Reading Frames

The recent development of ribosome profiling techniques enabled the discovery of many translationally active uORFs in the human genome [9]. These upstream reading frames can both produce proteins called uPeptides and have a regulatory role related to translation control of main ORF (mORF). It has also been shown that the most common start codons for uORFs are canonical AUG and non-canonical CUG, not AUG, AAG, and AGG as previously thought [10]. The functional changes in the AUG and CUG codons were demonstrated in samples from cancer patients, which may indicate their relationship with cancer biology [10]. The use of non-canonical start codons results from interactions with various trans-acting initiation factors and structural elements within mRNA [11]. In addition, translation initiation from non-canonical start codons results from, among others, stress conditions and may play key roles in the different regulatory mechanisms [12].

**Figure 1:**
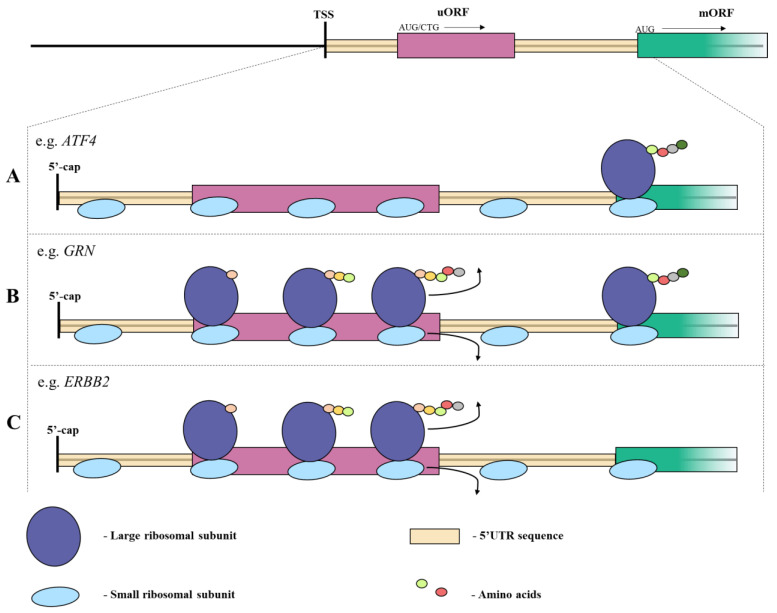
The most common uORF-mediated translation regulation pathways. (**A**) The leaky scanning phenomenon—uORF ribosome bypass without uProtein formation; (**B**) Production of uProteins by ribosomes, partial disassociation of the translational factors, and re-initiation of the mORF translation; (**C**) Production of uProteins by ribosomes, disassociation of the translational factors, and no translation of mORF.

Interestingly, not all uORF are translated into a protein (Figure 1A) and some have a regulatory function. An example is gene *ATF4*, whose expression is unregulated in response to stress, which is regulated by uORFs present in 5’UTRs. The mechanisms involved in *ATF4* regulation are called leaky scanning. In this phenomenon, ribosome bypass uORF without uProtein formation [13,14,15]. This results in a positive regulation of mORF translation by enhanced reinitiation after termination of uORF translation. The same uORF can be translated into uPeptide under stress-free conditions, down-regulating mORF expression [16,17,18]. uORF leaky scanning was observed for the *ATF4* gene in head and neck squamous carcinomas. It has been proven that up-regulation of the *DDX3* gene, whose product plays a regulatory role in translation, increases the translation of the *ATF4* mRNA through leaky scanning of its uORF. The ATF4 transcriptional factor positively influences the progression of neoplastic cells, enhancing their mesenchymal properties [19]. The mechanism of leaky scanning resulting in the enhanced translation of mORFs was also observed in the *Arabidopsis thaliana*. Under the hypoxia condition, the increased ribosome presence in the mOFRs regions was noticed, which results from the reduced uORF translation. Such mechanism may be a response to the challenging conditions [20].

Leaky scanning was also observed in non-stressful cell conditions. The mRNA of the *BACE1* gene consists of six uORFs, which inhibit or reduce the translation of the BACE1 protein. Deregulation of the inhibitory uORFs leads to increased *BACE1* expression, which was linked with the pathogenesis of Alzheimer’s disease [21,22]. Another example is the uORF located in the *APOBEC3G* mRNA that serves as repressor for mORF translation, regulating *APOBEC3G* expressions through leaky scanning, and re-initiation mechanism. Interestingly, the same uORF is used by the HIV-1 Vif protein to repress the *APOBEC3G* translation and redirect mRNA into stress granules. This repression is beneficial for the virus because the product of this gene inhibits its replication [23].

Another mechanism that regulates the translation of mORFs by uORFs is related to disassociation of non-essential factors. When non-essential factors disassociate after uORF translation e.g., the large ribosomal subunit, but the eIF3 stays on mRNA, the remaining factors necessary to initiate mORF translation may be re-recruited (Figure 1B) [24,25,26]. However, the efficiency of this re-recruitment depends on the distance between uORF and mORF [26]. An example of such regulation is the *GRN* gene which was identified to express transcripts with short and long 5′UTRs. An uORF within the long 5′UTR of *GRN* mRNA inhibits translation of the progranulin protein. It has been demonstrated that transcripts of the *GRN* gene with shorter 5′UTR do not contain an uORF, hence these shorter transcripts are not translationally repressed. The presence of the long 5′UTR is associated with a reduction of GRN levels [27]. This regulation mechanism was also observed in squamous cell carcinoma (SCC), where the uORF translation initiation of the *SOX2* gene is an important mechanism of tumor initiation properties [28]. Repressed expression of progranulin from long 5′UTR mRNA increases the risk for frontotemporal lobar degeneration (FTLD), which belongs to a group of progressive brain diseases [29]. Alternatively spliced 5′UTRs of the oncosuppressor *DLG1* represent another example on how different 5′ regions could impact gene expression and carcinogenesis. The longer *DLG1* 5′UTR contains an upstream short ORF, which inhibits translation of a downstream open reading frame [30]. It has been demonstrated that a higher expression level of transcripts with shorter 5′UTR results in an increased DLG1 protein level during epithelial junctions’ formation in colon cells. On the other hand, a higher amount of the larger mRNA isoform, causing a decrease in DLG1 protein level, was observed during monocyte-to-macrophage differentiation [31]. Moreover, the results of RT-qPCR analysis have shown up-regulation of the large 5′UTR *DLG1* mRNA isoform in cells with malignant potential [30]. Therefore, it is plausible that the mechanism of the 5′UTR splicing could control DLG1 protein abundance and potentially impact oncogenic processes by changing the *DLG1* levels [5].

uORFs as regulatory elements are able to control mORFs in a cis-acting manner, inter alia, via ribosomal subunits in eukaryotes [24,32]. The 43S preinitiation complex (PIC) PIC is made of many different proteins, including: eIF1, eIF1A, eIF2 ternary complex, eIF3, and 40S small ribosomal subunit [33,34,35]. The 43S PIC attaches to a 5′-cap structure of mRNA and slides the mRNA strand towards the 3′ end in search of a translation initiation codon [25,36]. Repression of mORF occurs when the critical translation initiation factors, e.g., eIF3 or eIF2, ternary complex disconnect after translating the uORFs and do not bind again downstream to initiate the translation of mORF (Figure 1C). For example, in *Saccharomyces cerevisiae*, the *NDC80* gene expresses two mRNA isoforms: the canonical *NDC80* mRNA isoform (*NDC80^ORF^*) and 5′-end-extended *NDC80* long undecoded transcript isoform (*NDC80^luti^*). In the elongated region, *NDC80^luti^* contains the regulatory uORFs that is blocking translation into the Ndc80 protein [37,38]. This mechanism was also observed in breast cancer, where the Her-2 receptor encoded by *ERBB2* gene is over-expressed compared to the non-cancer tissues. In non-cancer cells the Her-2 translation from mORF is repressed by the presence of uORF in 5’UTR. The over-expression of Her-2 in breast cancer cells is mediated by its 3’UTR interactions with uORF, blocking the formation of the uPeptide [39].

There is also a possibility that specific uORFs will repress the mORF translation through a particular peptide sequence [40,41]. The uORFs presence in the 5′UTRs may also affect the stability of mRNA through the nonsense-mediated mRNA decay (NMD) process [42,43]. However, not all uORFs can lead to NMD. Most often it concerns uORFs with canonical start codons. When translation can only be initiated from non-canonical start codons after canonical ones are eliminated, the resulting mRNAs are mostly not sensitive to NMD [44].

It has been shown that the genes in cancer cells have more uORFs than in healthy cells [45,46]. It has also been proven that mutations that arise within uORFs can lead to diseases in humans. An example is gonadal dysgenesis that is caused by the mutations in the uORF of the human *SRY* gene [47,48]. The presence of the uORF mutation in the human *IRF6* gene contributes to the emergence of the Van der Woude and popliteal pterygium syndromes [49] and in the human *GCH1* gene uORF can lead to symptoms of the Levodopa-responsive dystonia [50]. An interesting case of mutation in the 5’UTR of the human *HAMP* gene encoding hepcidin antimicrobial peptide was identified in the Portuguese Family. Mutation was associated with the appearance of a new translation start codon within the Kozak sequence. As a result, ribosomes translate the uORF protein and not the main hepcidin antimicrobial peptide, which leads to the hereditary hemochromatosis in young people [51]. A mutation identified in the 5′UTR of the *CDKN2A* tumor suppressor has a similar consequence and leads to translation from a novel AUG. It has been found that this mutation predisposes to melanoma [52]. Another example is an increased expression level of *TGF-β3* due to the G to A transition in the eleventh uORF. This mutation causes arrhythmogenic right ventricular cardiomyopathy/displasis (ARVC) [53]. An expansion of the GGC repeats in the 5’ UTR of the *N2C* gene leads to the translation of the uORF and emergence of the polyglycine rich uPeptide. This protein is accumulated in the cells intranuclear inclusions leading to the neuronal intranuclear inclusion disease [54]. In patients with pituitary adenoma and well-differentiated pancreatic cancer, a 4-bp deletion was identified within the 5’UTR sequence of the gene encoding the cyclin-dependent kinase inhibitor p27 (KIP1)—*CDKN1B*. The role of this atypical tumor suppressor is to regulate the cycle, proliferation, and differentiation of the cell. The above-described deletion is located within the uORF sequence and causes a stop codon shift. Consequently, a longer uPeptide is formed and the distance to the mORF is shortened, which leads to a decrease in the CDKN1B and p27 protein level [55]. It has also been shown that the loss of the start codon by uORF of the *CEBPB* gene leads to the formation of two short forms of proteins: C/EBPα and C/EBPβ. Moreover, increased levels of the short form of C/EBPα have been associated with acute myeloid leukemia and C/EBPβ with breast cancer [56].

Peptides resulting from the translation of uORF can also play a regulatory role in various processes. It has been shown that a uPeptide called MP31 arising from the uORF of the *PTEN* gene limits the lactate-pyruvate conversion process taking place in mitochondria [57]. Another example is a uORF of protein kinase C-eta (PKC-η) mRNA that encodes a peptide (uPEP2). This peptide exhibits the kinase inhibitory activity, which through typical protein kinase C pseudo-substrate motif auto-inhibits the catalytic kinase activity of all members of the protein kinase C family. In consequence, uPEP2 positively affects chemotherapy of breast cancer through inhibiting the cancer cell proliferation, survival, invasion, and metastasis [58]. There are also other examples of peptides generated from uORF located in 5′ UTRs of genes, including: *UL4*, *TRPM7*, *DDIT3*, *PPP1R15A,* and *PTP4A-2* [24,59].

## 3. Head-to-Head Overlapping Genes Phenomenon

Genes that sequences partially or completely share the locus on the same or opposite DNA strand are defined as overlapping genes [7]. The first overlapping genes were already identified in 1969 in the coliphage genome [60] and soon were found to be common in viral and bacterial genomes [61]. However, until the beginning of this century, there was limited evidence of the existence of overlapping protein-coding genes in eucaryotic genomes. Nevertheless, multiple studies have revealed that this phenomenon is more common than previously thought. Overlapping genes have been detected in many organisms such as plants [62], yeast [63], fish [7], flies [64], mice [65], and humans [66,67]. Based on the recent analysis, Chen et al. claimed that 25.8% of the human protein-coding genes overlap [68]. Ho et al. identified 2541 overlapping gene pairs in the human genome. Among them, 473 pairs overlap in the head-to-head orientation (i.e., with their 5′ ends) [69]. Results of bioinformatics research conducted on samples from 73 human tissues and cell lines revealed that from a total of 15,778 of protein-coding genes, 582 pairs overlap at their 5′ ends [70].

The majority of 5′ overlapping genes were identified based on bioinformatics analyzes. However, some were discovered experimentally. Two examples are human *DLG4* and *VLCAD* genes. The 5′-untranslated region of *DLG4* overlaps with the entire 5′UTR and 62 bp of the coding sequence of *VLCAD*. Despite overlapping at the 5′ ends and sharing regulatory components, both genes are highly expressed at the same time in various tissues [71]. Overlapping genes could show tissue-specific expression patterns [72,73], co-expression [71], and co-regulation [74,75]. Some studies suggest that coordinated gene expression is common for genes located on opposite strands and overlapping in the 5′UTRs [76]. On the other hand, there are also works showing that the phenomena of gene overlap could lead to promoter competition and a negative expression correlation of involved genes [69,73]. However, recent studies demonstrated that this genomic arrangement may lead to a higher expression level of at least one gene from overlapping pair [70,77].

In the majority of cases, analysis of the human transcriptome have shown that an overlap between two genes located on opposite DNA strands is not a stable feature and depends on which alternative transcription start site (TSS) is activated. The utilization of more distal TSS causes an extension of the 5′UTR, resulting in genes’ overlapping [70]. As an example, when simultaneously utilizing alternative TSSs, human genes *FBXL15* and *PSD* were identified to overlap at their 5′ ends in brain tissue. The overlap between these genes occurs only when distal TSSs are used [78]. The overlap at the 5′ ends is thought to be involved in various regulatory events, such as transcriptional interference [79] and RNA:RNA duplex formation [80]. However, despite the number of studies, the biological meaning of this genomic architecture is still debatable.

## 4. RNA Duplexes

Owing to sequence complementarity transcripts of overlapping genes may form RNA:RNA duplexes, which can affect transcription and translation of involved genes [80]. The formation of RNA:RNA duplexes may interfere with the alternative splicing process [81], cover miRNA-binding sites [82,83], or influence mRNA translation [84]. Kudla et al., have proved that the blockage of mRNA 5′UTR in *E. coli* results in poor translation efficiency [85]. However, the detection of RNA:RNA duplexes is technically difficult, mainly due to the instability of double-stranded RNA in eukaryotic cells. An additional complication in the identification of mRNA duplexes comes from the fact that this RNA:RNA hybrid can trigger a process leading to cutting double-stranded RNA into short duplexes—precursors of endo-siRNA [86,87]. It has also been demonstrated that the currently used methods have too low of a sensitivity and specificity to allow accurate identification of duplexes. So far, only a few RNA:RNA duplexes between two protein-coding transcripts have been experimentally validated. The *Wrap53* and *p53* gene pair is one of the best-characterized examples, for which RNA duplexes formation was confirmed in human cell lines [88].

**Figure 2:**
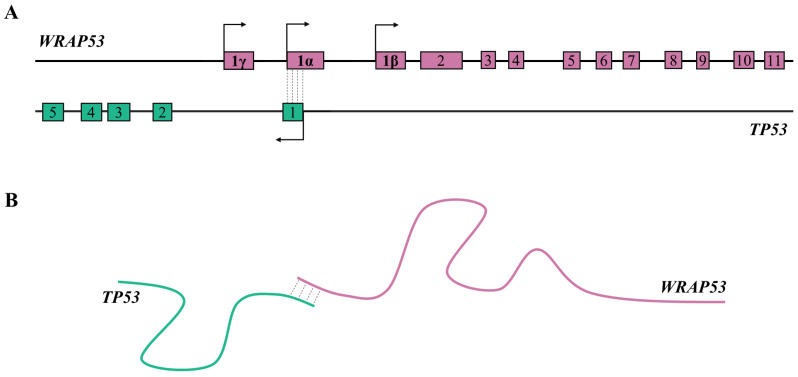
The *WRAP53* and *TP53* genes (**A**) and its RNAs duplex formation (**B**). The dotted lines demonstrate overlapping region between these genes (**A**) or created RNA duplex (**B**) and the arrows show the direction of transcription. Numbered regions indicate the coding sequences/exons of the *WRAP53* and *TP53* genes.

A key tumor suppressor gene, *p53*, is a transcription factor whose function has been identified in numerous biological processes, including metabolism, senescence, or cell cycle arrest and apoptosis in response to cellular stress [88,89,90]. To avoid this response, *p53* is often inactivated in cancer cells, allowing survival and tumor progression [89]. The identification and characterization of natural antisense transcript *Wrap53* positioned opposite to the *p53* DNA strand revealed that both genes overlap at the 5′ ends (Figure 2A) [88]. The *Wrap53* termed for WD40-encoding RNA antisense to p53 encodes the WRAP53 protein (also denoted WDR79), crucial for cellular trafficking of small Cajal body-specific RNAs (scaRNAs) and recruitment of the telomerase enzyme to Cajal bodies [91,92,93]. *Wrap53* utilizes three alternative transcriptional start sites (1α, 1β, and 1ɣ) [92]. Usage of distal TSS (1α) results in the elongation of 5′UTR of the transcript described as WRAP53-1α. WRAP53-1α directly overlaps the first exon of the *p53* gene and regulates p53 mRNA at the transcriptional level [88]. Studies revealed that both genes interact at the 5′ ends, increasing the stability of *p53* mRNA and enhancing the production of the p53 protein in response to cellular stress [88,94]. It has been found that *Wrap53* regulates *p53* via RNA:RNA hybridization (Figure 2B). Inhibition of *Wrap53/p53* duplex formation reduces *p53* abundance, confirming that this RNA:RNA interaction protects *p53* from degradation and sustains its expression in human cells [88]. Since the *p53* expression level is regulated by *Wrap53,* any alterations of *Wrap53* levels could contribute to tumorigenesis [92]. Over-expression of *Wrap53* has been associated with several types of tumors, including head and neck squamous cell carcinoma (HNSCC) [94,95], development of esophageal squamous cell carcinoma (ESCC) [96], or tumor progression in non-small cell lung cancer (NSCLC) [97].

Recent studies have shown that two transcript isoforms of the *Wrap53* gene have distinct and opposite functions in cancer cell lines. Elongated at 5′UTR isoform WRAP53-1α regulates *p53* expression, while the shorter transcript WRAP53-1β has no regulatory effect on p53 [98]. However, WRAP53-1β plays an important function in the repair of DNA double-strand breaks [99]. It has been shown that knockdown of WRAP53-1α suppresses cell migration in the A549 cell line, whereas depletion of WRAP53-1β promoted cell migration in these cells. Moreover, WRAP53-1α deficiency promoted H1975 cell invasion, but knockdown of WRAP53-1β had no significant effect on H1975 cells. Altogether, these results suggest that compared to the shorter isoform, the 5′UTR elongated transcript of *Wrap53* has different effects on *p53* and NSCLC cells [98].

Several consequences of the 5′-ends interaction between *p53* and *Wrap53* have been shown over the years. Interestingly, bioinformatics research revealed that in breast cancer the part of 5′UTR of *Wrap53* mRNA that overlaps the *p53* transcript contains a binding site for has-miR-4732-5p. Therefore, has-miR-4732-5p miRNA by binding to 5′UTR of the *Wrap53,* may block *Wrap53/p53* duplex formation and disturb the *p53* level [83].

There are some other instances of the regulatory role of RNA:RNA duplexes formed by two mRNA sequences. In human cells, splicing of *TRα1* is regulated via mRNA-mRNA interactions with *Rev-erbα* [100]. Moreover, in gastric cancer, human genes *WDR83* and *DHPS* regulate and increase their mutual stability via the formation of a RNA:RNA duplex [101]. An example also comes from *Arabidopsis thaliana,* where formation of RNA duplexes with regulatory consequences was detected for *POR1-OCA2* [102] and *SRO5-P5CDH* gene pairs [103].

Approaches focusing on identifying RNA duplexes in living cells have been significantly improved over the past years. More and more evidence for duplexes formed by protein-coding genes transcripts is coming from high-throughput techniques. Sharma et al. described the LIGR-seq method, which allows the study of global-scale RNA:RNA interactions in vivo. However, the yield of RNA–RNA interaction detected in this study was very low and constituted only 1029 reads [104]. In more precise methods called PARIS [105,106], 232,031 reads of RNA–RNA interactions were obtained but it is unknown how many of them were formed by two mRNAs [107]. A high-throughput method named SPLASH resulted in 4026 reads, including 990 mRNA–mRNA interactions in human cell lines [108]. All these methods enable global RNA interactome analysis but still have to be refined and new approaches need to be developed.

## 5. Transcriptional Interference

The phenomena of overlapping genes could lead to the downregulation of both genes via transcriptional interference mechanisms. TI occurs when one transcriptional process inhibits another transcriptional process taking place on the opposite DNA strand at the same time [79]. Over the past few years, the regulation of gene expression through transcriptional interference has been confirmed in various organisms, from viruses to metazoans [109]. Mechanisms of TI have been observed in *Escherichia coli* [110] and *Saccharomyces cerevisiae* [111]. Transcriptional interference has been also identified to play an important role during embryonic development in *Drosophila melanogaster* [112].

Four main mechanisms of transcriptional interference have been defined: promoter competition in the initiation phase of transcription, polymerase collisions, ‘sitting duck’ interference, and occlusion in the elongation phase of transcription [79]. Promoter competition occurs when promoters of head-to-head overlapping genes compete for the RNA polymerase II (RNAP II) complex [79]. In eukaryotes, promoter competition also happens when two promoters of overlapping genes share the same transcriptional factors binding sites. When these factors bind to the enhancers of one promoter, the second promoter enhancers are prevented from interacting and activating [113]. The mechanism of polymerase collision occurs in the elongation phase when the RNAP II complex from one strand acts as a physical barrier and prevents the RNAP II complex on the other strand from progressing [114,115]. In the head-to-head overlapping region, two proceeding polymerase complexes collide, resulting in untimely transcription termination and a decrease in the expression level of both involved genes [79,80]. According to the results of a bioinformatics study, the longer the overlap region, the greater the probability of RNAP II complexes collision [114]. ‘Sitting duck’ interference is considered when the weaker promoter’s RNAP II complex slowly progresses from the open complex to the extension complex and might be pushed out by the elongation complex from the stronger, convergent promoter [79]. The elongation of RNA polymerase may also inhibit transcription from the downstream promoter in a process described as promoter occlusion [116]. An extending RNAP II launched from the tandem promoter temporarily occupies the downstream promoter and its activator binding sites. As a result, the time in which RNAP II can bind to the downstream promoter is limited [79]. Transcriptional interference could lead to a negative expression correlation of overlapping genes [69,73]. However, recent studies have demonstrated that the utilization of overlapping TSSs may lead to, on average, higher genes expression [70,77]. Studies performed by Rosikiewicz et al. [70] on TSS-seq data from 73 human tissues and cell lines also showed that genes overlapping at the 5′ ends do not have a negative expression correlation.

Transcriptional interference is not limited to overlapping genes. Tandem transcriptional interference applies to the situation when the process of transcription from an upstream promoter inhibits the transcription of a co-oriented downstream promoter [117]. Brar et al., have shown that in budding yeast over 190 genes produce 5′UTR elongated mRNA isoforms. It has been confirmed that in some of them the expression of 5′ extended mRNA inhibits the expression from the downstream promoter [118,119]. For example, in *Saccharomyces cerevisiae*, TI occurs during cell differentiation and regulates the expression of the *NDC80* gene [37]. The *NDC80* gene encodes a subunit of the Ndc80 outer kinetochore complex, which is required for the connection of spindle microtubules to kinetochores during yeast meiosis [38,120]. In meiotic cells, the *NDC80* gene expresses two mRNA isoforms. During the prophase stage of meiosis, the expression of the canonical *NDC80* mRNA isoform (*NDC80^ORF^*) is repressed by the transcription of a 5′-end-extended *NDC80* long undecoded transcript isoform (*NDC80^luti^)* [37,38]. The transcription of a 5′ elongated *NDC80* mRNA isoform provides a repressive chromatin state and leads to transcriptional interference of a downstream promoter of *NDC80^ORF^*. Results of these studies demonstrate that the 5′UTR elongated *NDC80* transcript isoform plays a regulatory role and its expression impacts the cell by causing transcriptional interference at the *NDC80^ORF^* promoter during meiotic prophase [37]. Since transcripts with elongated 5′UTR are also expressed in higher eukaryotes transcriptional interference caused by 5′UTR, elongated isoforms might be widespread across species [37]. Interestingly, in human prostate cancer cells, the constitutively activated upstream promoter of the *ATF3* gene leads to a higher expression of *ATF3* in stress response, while expression from the downstream promoter is suppressed [121]. It is proposed that the upstream promoter of the human *ATF3* gene could inhibit the transcription from the downstream promoter due to delayed transcriptional interference [79,106].

## 6. 5’UTR Sequences as Potential miRNA and Protein Binding Sites

### 6.1. 5′UTRs and Interactions with miRNAs

The regulation of gene expression by the presence of miRNA binding sites in mRNAs is well known [122,123,124]. Most of the interactions between miRNAs and mRNAs are related to the 3’UTRs, which has been confirmed by many studies [125,126,127,128]. However, there is strong evidence of a major role for the 5’UTR sequence in miRNA drive regulation of expression [129]. It has also been shown that structures present at the 5’-end of transcripts may influence interactions with miRNAs and the higher the degree of secondary structures in 5’UTR fragment, the greater the chance of these interactions. The big importance of secondary structures close to the 5’ cap site on the formation of interactions between the miRNA and the 5’UTRs has been also demonstrated [130].

There is evidence for the positive miRNA regulation of mRNAs encoding ribosomal proteins (RP). miR-10a by joining with the 5’UTR sequence of RP mRNAs enhances the synthesis of ribosomal proteins positively influencing their biogenesis. It has been shown that most RP mRNAs regulated in this way had a 5’TOP motif associated with the presence of C residues at the sequence beginning [131]. Another example comes from colorectal cancer (CRC), where miRNA—miR-532-5p is involved in the process of carcinogenesis. Increased expression of this miRNA has been associated with a decreased expression of the runt-related transcription factor 3 (*RUNX3*) gene in CRC. Studies conducted on human HT—29 CRC cells have shown that miR-532-5p binds specifically to the 5’UTR region of the *RUNX3* mRNA, which in turn increases the viability and proliferation of cancer cells, positively influencing their expansion processes [132].

There is also evidence for miRNA targeting 5′UTRs in viruses. An interesting mechanism was noticed during the replication of the hepatitis C virus (HCV). This virus has a positive (+) strand RNA. In the 5’UTR region of this viral sequence, there are two hepatic-specific miR-122 binding sites. The attachment of this miRNA to both binding sites within the 5’UTR of the viral genome enables up-regulation of replication [133]. Several binding sites for different miRNAs have also been described in the 5’UTR of SARS-CoV-2, suggesting that also here they may have a regulatory function [134,135].

### 6.2. 5′UTRs and Interactions with RNA Binding Proteins

The 5′UTR sequence has a critical role in the recruitment of ribosomes to mRNA as well as in many processes related to the mechanisms regulating translation. Translation control by 5’UTR may result from direct induction or by obliterating the ability to bind to RNA binding proteins (RBPs). An example of translation control through 5’UTR-protein interactions is the *STAT3* mRNA translation inhibition through poly(rC)-binding protein 1 (PCBP1). Two leucine residues of the PCBP1 protein bind to *STAT3* 5′UTR and, by reducing the amount of oncogenic protein STAT3, serve as a tumor suppressor [136]. Another study has shown that translation of the *ELAVL4* gene is associated with alternative 5′UTR sequences of the distinct mRNA isoforms in neuronal development. This translation is regulated by RNA binding protein—Celf1 in the development of glutamatergic neurons. Dysregulation of these mechanisms can lead to neurological disorders and tumor formation [137]. In *Caenorhabditis elegans*, the LIN41 protein represses translation of the *lin-29A* gene through interaction with the 5′UTR of *lin-29A* mRNA. It has been suggested that the attachment of LIN41 to the 5’UTR sequence of the *lin-29A* gene mediates mRNA degradation by recruiting other proteins, including ribonucleases [138]. There is evidence that some RBP may act differently on UTR sequences in a position-dependent manner by binding to the 5’UTRs or 3’UTRs. Examples of this mechanism are metazoan iron regulatory proteins (IRPs). When they interact with the 5′UTRs, they inhibit mRNA translation, while when they bind to the 3’UTRs sequence, they increase the stability of the mRNA, making it less prone to degradation [139]. A similar mechanism was also observed in *Drosophila melanogaster*, where in female flies there is a need for *MSL-2* mRNA translation inhibition. The Sex-lethal (SXL) protein binds both 5′- and 3′UTR. When SXL binds to the *MSL-2* 3′UTR sequence it inhibits the attachment of 43S ribosomal preinitiation complexes and when it binds to the 5′UTR sequence, it blocks the ribosomal complexes scanning for the translation initiation codon of mRNA [140].

The 5′UTRs contain internal ribosome entry sites (IRESs) that enable the binding of the 40S ribosomal subunits to the mRNA [141]. Therefore, mutations in IRES could impact translation and as a result various diseases [142,143]. In the nervous system, the C to T transition in *Connexin-32* mRNA leads to the loss of IRES-mediated translation of *Connexin-32.* This mutation is responsible for the development of X-linked Charcot–Marie–Tooth disease (CMTX) [144]. It has also been reported that mutation in the IRES of the *c-Myc* mRNA enhanced its translation and influenced multiple myeloma progressions [145,146].

The 5′UTR sequence has its role in the regulation of mouse insulin biosynthesis. Proteins PABP, HuD and Protein-Disulfide Isomerase are involved in the regulation of glucose level. In the low/basal glucose conditions the PABP and HuD form a translation inhibitory complex by binding to the 5′UTR of the *Ins2* gene mRNA. When the glucose level rises, this complex is affected by Protein-Disulfide Isomerase, causing insulin translation to be stimulated [147].

The regulatory role of 5’UTRs in translation may also involve formation of secondary structures. L-ferritin mRNA contains a stem-loop structure IRE within 5′UTR. Disruptions of the IRE inhibits its interaction with iron-regulatory proteins, resulting in abnormal L-ferritin production. Changes in L-ferritin levels have been reported in the hereditary hyperferritinaemia/cataract syndrome (HHCS) [148,149]. In colorectal cancer, the cellular inhibitor of PP2A—CIP2A protein regulates translation of the *MYC* gene mRNA and by increasing the level of MYC protein leads to carcinogenesis. A suggested explanation for this phenomenon is the presence of the IRES elements and G-quadruplexes structures in the 5′UTR sequence of *MYC* mRNA [150]. The G-quadruplex structure is another regulatory element that is frequently observed in 5′UTR sequences. They have been revealed by various laboratory techniques that include the following: antibody arrays and RGB-1 coupled with small molecules [151], SHALiPE structural analysis [152], crystallization using iSpinach aptamer fluorescent complex with the 3,5-difluoro-4-hydroxybenzylidene imidazolinone (DFHBI) [153], and luminescent-based techniques [154]. The structure of G-quadruplex was identified in the 5′UTR sequence of the hepatocyte nuclear factor 4-alpha (*HNF4α)* gene. The inhibition of the HNF4α translation through interaction between 5′UTR G-quadruplex and RNA binding proteins can lead to carcinogenesis in the human liver [155,156]. Another mechanism based on the 5′UTR secondary structure was observed in human skeletal muscles. The DHX36 helicase is responsible for unwinding the G-quadruplex structures. It binds to the 5′UTR secondary structure of the *GNAI2* gene mRNA. When DHX36 rewinds the G-quadruplex structure, the Gnai2 protein is up produced in muscle resident stem cells. This induces proliferation and thus increases the ability of these cells to regenerate [157].

## 7. 5’UTRs and Their Other Implications

As was described above, transcripts with alternative 5′-untranslated regions, by affecting mRNA stability and translational efficiency, can determine gene expression [158,159]. These mRNA variants may also differ in the length of 5′ untranslated regions (UTRs) [160,161], display distinct expression patterns [162], and have various and even opposite biological functions [98,163]. By expression in a tissue-specific manner, these transcripts may regulate protein expression and control developmental and physiological processes [159].

The deregulation of translation, via the 5′UTR region, is associated with many diseases, including cancers [164,165,166]. In fact, long and complex 5′UTRs are more frequently expressed by oncogenes and tumor suppressor genes [167]. *BRCA1*, *Mdm2*, and *AXIN2* are just a few examples of cancer related genes that utilize alternative 5′UTRs [159,168,169].

The tumor suppressor gene *BRCA1* is involved in breast and ovarian cancer development, mainly through decreased levels of *BRCA1* mRNA. Two *BRCA1* transcripts that use alternative promoters show distinct expression patterns [159,168]. Transcript with longer 5′UTR is expressed only in breast cancer tissue, whereas *BRCA1* mRNA with shorter 5′UTR has been found in cancerous and noncancerous breast tissues [168]. Elongated at the 5′ end mRNA of the *BRCA1* gene has a more complex secondary structure and upstream AUG codons, which have been shown to reduce translational efficiency compared to a shorter isoform [161,170]. Deregulated expression of the *BRCA1* increases the amount of 5′ extended transcript and causes a decrease in the BRCA1 protein noticed in breast and ovarian cancers [168].

The *BCRP* gene that encodes breast cancer resistance protein also expresses mRNAs with different 5′UTRs. *BCRP* is frequently over-expressed in human cancers and leukemia, causing multidrug resistance in chemotherapy [164,171]. It has been found that *BCRP* gene utilizes tissue-specific promoters that produce at least three 5′UTR variants. The expression level of the *BCRP* mRNA isoform with the longest 5′UTR has been increased in the drug-resistant cells. In human breast cancer, cell lines selected with Adriamycin and verapamil (MCF-7/AdrVp) transcripts with extended 5′UTR constituted 47%; in ovarian carcinoma, cells selected with topotecan (Igrov1/T8) 71%; and with mitoxantrone (Igrov1/MX3) cell, 66% of the total *BCRP* mRNA transcripts, implying that this isoform is predominant in drug-resistant cells. Furthermore, mRNA containing longer 5′UTR is more efficiently translated compared to isoforms with shorter 5′UTR, thus can regulate BCRP protein levels [164]. The high expression level of *BCRP* is also associated with a poor prognosis for acute myeloid leukemia (AML) patients [172]. Results of analysis of the *BCRP* transcripts isoforms from pediatric AML samples revealed that a novel 5′UTR located 90 kb upstream of the exon 2 translation initiation site was expressed in 40% of the bone marrow samples and in all acute megakaryoblastic leukemia (AML FAB-M7) cases. Interestingly, expression from this upstream promoter wasn’t detected in non-hematopoietic cell lines. These findings suggest that *BCRP* utilizes a tissue-specific promoter and high expression of transcripts isoforms generated from this promoter may have a negative impact on M7 AML subtype [173].

The expression level of the ERβ protein is strongly influenced by the expression of alternative 5′UTRs. ERβ is estrogen receptor β, which regulates cell growth and differentiation. Studies have demonstrated that two alternative 5′UTRs (longer UTRa and shorter UTRb) of *Erβ* have tissue-specific distribution. Additionally, evidence implies that 5′UTRs may determine downstream splicing events, potentially influencing ERβ function [167,174].

An activating transcription factor 3 (ATF3) determines cell fate due to the regulation of stress response and its expression was increased in the human prostate [175]. The *ATF3* alternative promoter described as P1 was identified ~43.5 kb upstream of the P2 promoter. Multiple transcriptional start sites and various 5′UTRs of the P1 transcripts were detected. Transcripts with the longest 5′UTR contain inhibitory elements, whereas the stimulatory elements are present in the shorter 5′UTRs. These features are especially important in response to serum or oxidoreductive stress. Furthermore, results suggest that the upstream P1 promoter is constitutively activated in human cancer cells [121].

## 8. Conclusions

The 5’UTR sequences via different mechanisms play an important role in the regulation of both transcription and translation. Some of these mechanisms enable the proper functioning of the cell, while others lead to pathological changes, which are summarized in Table 1. Many of these mechanisms have been well understood and explained, while others require further investigation and clarification. With the constant development of laboratory techniques, such as ribosome profiling, crystallization, and genome/transcriptome editing tools, we can expect a more thorough elucidation of all the mechanisms involving 5’UTRs in near future.

## Figures and Tables

**Table 1:** List of genes that various regulatory elements of the 5′UTR are involved in diseases.

Regulatory Elements	Gene	Disease	References
De-regulation of uORFs	*BACE1*	Alzheimer’s disease	[21,22]
	*ATF4*	Head and neck squamous carcinomas	[19]
	*Her-2*	Breast cancer	[39]
	*SOX2*	Squamous cell carcinoma (SCC)	[28]
	*CDKN2A*	Melanoma	[52]
	*SRY*	Gonadal dysgenesis	[47,48]
	*IRF6*	Van der Woude and Popliteal Pterygium Syndromes	[49]
	*GCH1*	Levodopa-responsive dystonia	[50]
	*HAMP*	Hereditary hemochromatosis	[51]
	*N2C*	Neuronal intranuclear inclusion disease (NIID)	[54]
	*CDKN1B*	Pituitary adenoma, Pancreatic cancer	[55]
	*CEBPB*	Acute myeloid, Breast cancer	[56]
	*EPHB1*	Breast cancer, Colon cancer	[46]
	*MAP2K6*	Colon adenocarcinoma	[46]
	*TGF-* *β3*	Arrhythmogenic right ventricular cardiomyopathy/dysplasia (ARVC)	[53]
	*DLG1*	High risk of human papillomavirus (HPV)	[5,30]
Length of 5′UTR	*BRCA1*	Hereditary breast-ovarian cancer syndrome	[159,168]
	*BCRP*	Breast cancer	[164]
		Acute myeloid leukemia (AML)	[173]
	*ERβ*	Breast and lung cancer	[167,174]
	*GRN*	Progressive brain diseases	[27]
	*ATF3*	Prostate cancer	[121]
IRES	*Connexin-32*	X-linked Charcot–Marie–Tooth disease (CMTX)	[144]
	*c-Myc*	Multiple myeloma patients	[145,146]
miRNA and protein binding sites	RUNX3	Colorectal cancer	[132]
	STAT3	Several types of tumors	[136]
	ELAVL4	Neurological disorders	[137]
Secondary and stem-loop structures	L-ferritin	Hereditary hyperferritinemia/cataract syndrome (HHCS)	[148,149]
	MYC	Colorectal cancer	[150]
	HNF4α	Liver cancer	[155,156]

## Data Availability

Not applicable.
